# Detection of bovine viral diarrhea virus genotype 1 in aerosol by a real time RT-PCR assay

**DOI:** 10.1186/s12917-020-02330-6

**Published:** 2020-04-15

**Authors:** Peili Hou, Yaru Xu, Hongmei Wang, Hongbin He

**Affiliations:** Ruminant Diseases Research Center, College of Life Sciences, Shandong Normal University, No.88 East Wenhua Road, Jinan City, Shandong Province China

**Keywords:** Bovine viral diarrhea virus (BVDV), Real-time RT-PCR, BVDV-1 aerosol, Detection

## Abstract

**Background:**

As a *pestivirus* of the *Flaviviridae* family, bovine viral diarrhea virus (BVDV), has imposed a large burden on animal husbandry worldwide, and such virus can be transmitted mainly through direct contact with other infected animals and probably via aerosols. In the present study, we aimed to develop a real-time RT-PCR method for detection of BVDV-1 in aerosol samples.

**Methods:**

A pair of primers specific for highly conserved regions of the BVDV-1 5′-UTR was designed. The standard curve and sensitivity of the developed assay were assessed based on 10-fold serial dilutions of RNA molecular standard. The specificity of the assay was evaluated with other pestiviruses and infectious bovine viruses. The clinical performance was examined by testing 169 aerosol samples.

**Results:**

The results showed that a good linear relationship existed between the standard curve and the concentration of template. The lowest detection limit was 5.2 RNA molecules per reaction. This assay was specific for detection of BVDV-1, and no amplification was found for other pestiviruses such as classical swine fever virus (CSFV), border disease virus (BDV), and common infectious bovine viruses, including BVDV-2, infectious bovine rhinotracheitis virus (IBRV), bovine parainfluenza virus type 3 (BPIV-3), bovine respiratory syncytial virus (BRSV), bovine ephemeral fever virus (BEFV) and bovine coronavirus (BcoV). The assay was highly reproducible with low variation coefficient values (CVs) for intra-assay and inter-assay. A total of 169 aerosol samples collected from six dairy herds were tested using this method. The results showed that the positive detection rate of BVDV-1 was 17.2% (29/169), which was significantly higher compared with the conventional RT-PCR. Additionally, the positive samples (*n* = 29) detected by real-time RT-PCR were verified by BVDV RPA-LFD, and a concordance rate of 100% was obtained between them.

**Conclusions:**

Taken together, we developed a real-time RT-PCR assay for quantitative analysis of BVDV-1 in aerosol samples, and our finding provided valuable insights into the risk on aerosol transmission of BVDV-1.

## Background

Bovine viral diarrhea virus (BVDV) is a causative agent of numerous clinical symptoms in cattle, including diarrhea, hemorrhagic syndrome, reproductive and respiratory disorders, persistent infections, and mucosal disease, leading to significant financial losses to the global livestock industry [1–3]. Transmission of BVDV occurs both horizontally and vertically since infectious virus particles are shed by persistently and transiently infected animals via direct contact, bodily secretions and contaminated fomites [4, 5]. As BVDV is able to persist in the environment for more than 2 weeks, airborne transmission is one of the potentially important routes for the spread of BVDV, especially in the majority of herds with a high density of stocking cattle [6].

BVDV, classical swine fever virus (CSFV) and border disease virus (BDV) belong to the genus *Pestivirus* of the *Flaviviridae* family. The genome is a single-stranded, positive-sense RNA molecule consisting of a 5′ untranslated region (5′-UTR), a single open reading frame (ORF) encoding structural proteins and non- structural proteins, and a 3′-UTR [7]. The 5′-UTR is most often targeted for molecular diagnosis technology and genotyping since it is highly conserved in the pestivirus genome [8–11]. Commonly, BVDV strains can be categorized into BVDV-1 and BVDV-2, and each genotype has been further divided into distinct subtypes [12–15]. Although both genotypes have been diagnosed worldwide, the prevalence of subtypes geographically varies, and BVDV-1 has the highest occurrence in cattle population in China [16, 17]. Therefore, rapid diagnosis of BVDV-1 epidemic strains has important significance in epidemiological studies, vaccine development, and disease management.

Different strategies, such as bulk tank milk (BTM) serology can be used to monitor the status of herd infection. However, BTM has limitations as a diagnostic material for BVDV detection since antibody response in infected animals can still be detected in serum and milk long after the virus is no longer present [18, 19]. Aerosol is a relatively stable dispersion system suspended in a gaseous medium by solid or liquid particles, and the size of those particles generally ranges from 0.001 μm to 100 μm in diameter. Detection of aerosol is also an efficient way to monitor infectious diseases in dairy herds for larger-scale epidemiological investigation [20–22]. At present, the study of bioaerosol mainly focuses on the detection of airborne bacteria, fungi and endotoxin [23–25]. However, viruses from bioaerosol particles have relatively lower concentrations compared with other microorganisms in ambient air, and ultrafine virus aerosols make them difficult to collect for study. Furthermore, the airborne route of BVDV infection has not been well documented. Therefore, the lower contamination rate and specificity of molecular techniques present a good technical model to further study the aerosol transmission of BVDV infection. SYBR Green I-based quantitative RT-PCR assay is the simplest and most economical molecular detection method, which has various advantages over the Taq-Man assay, including low cost, easy design and testing of primers without fluorescent probes, and simple standardization of experiments. Additionally, it is insensitive to nucleotide variations of highly mutating RNA viruses that occur within the fluorescent probe based target region, leading to lower false-negative results [26, 27].

In the present study, we aimed to develop a SYBR Green-based real-time RT-PCR approach with an acceptable sensitivity, specificity and reproducibility. Moreover, we also evaluated its performance on detection of BVDV-1 in aerosol samples collected from different herds of dairy cattle.

## Results

### Standard curve, sensitivity, and melting temperature (T_m_) profile of the real-time RT-PCR

**Fig. 1:**
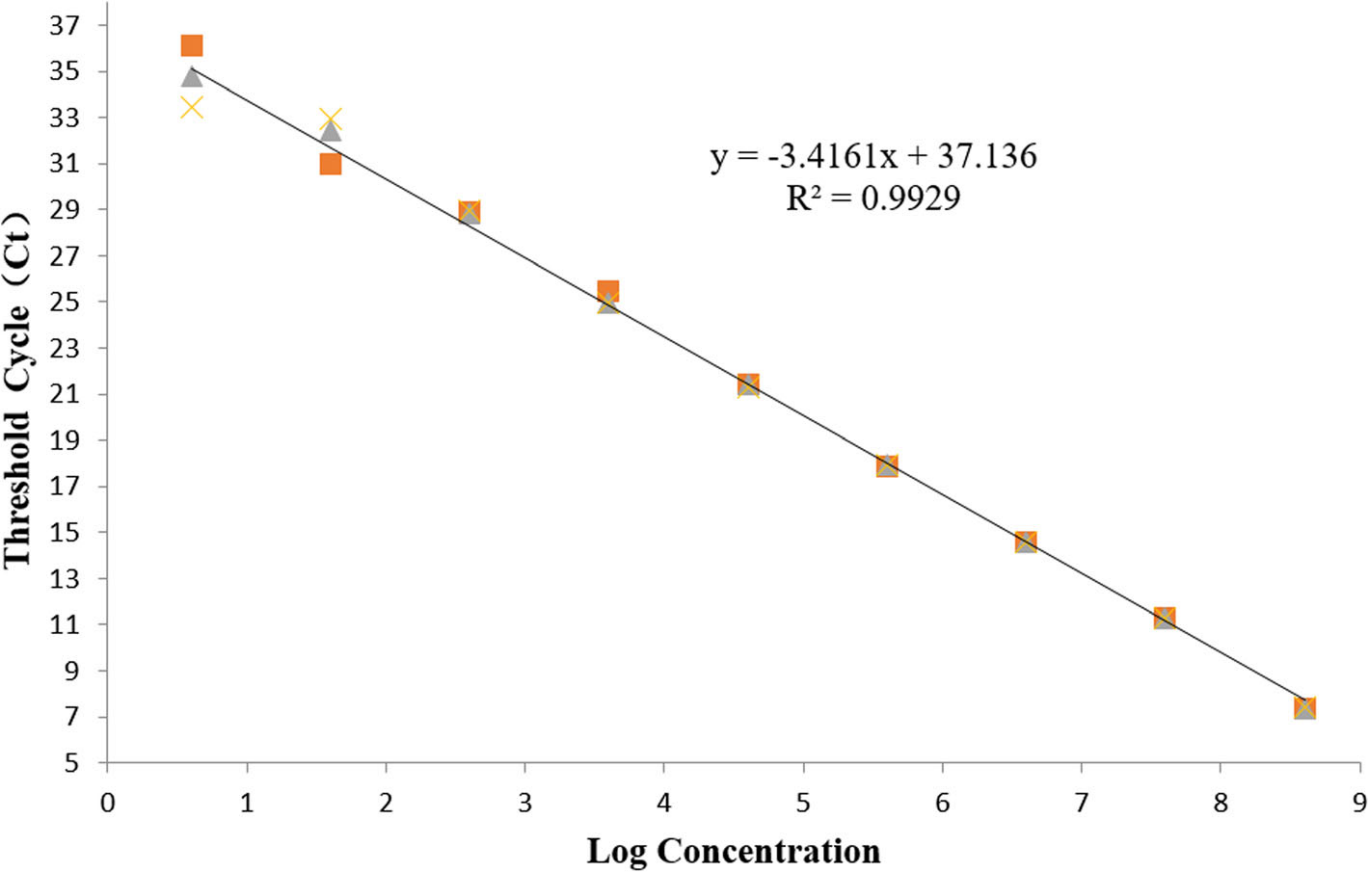
Standard curve generated by real-time RT-PCR. The dilutions of RNA molecular standard concentrations (Log) are indicated on the x-axis, whereas the corresponding Ct values are presented on the y-axis. Each dot represents the result of duplicate amplifications of each dilution. The coefficient of determination (R^2^) and the slope value (s) of the regression curve were calculated and indicated as log RNA molecules (5.2 × 10^8^–5.2 × 10^0^)

**Fig. 2:**
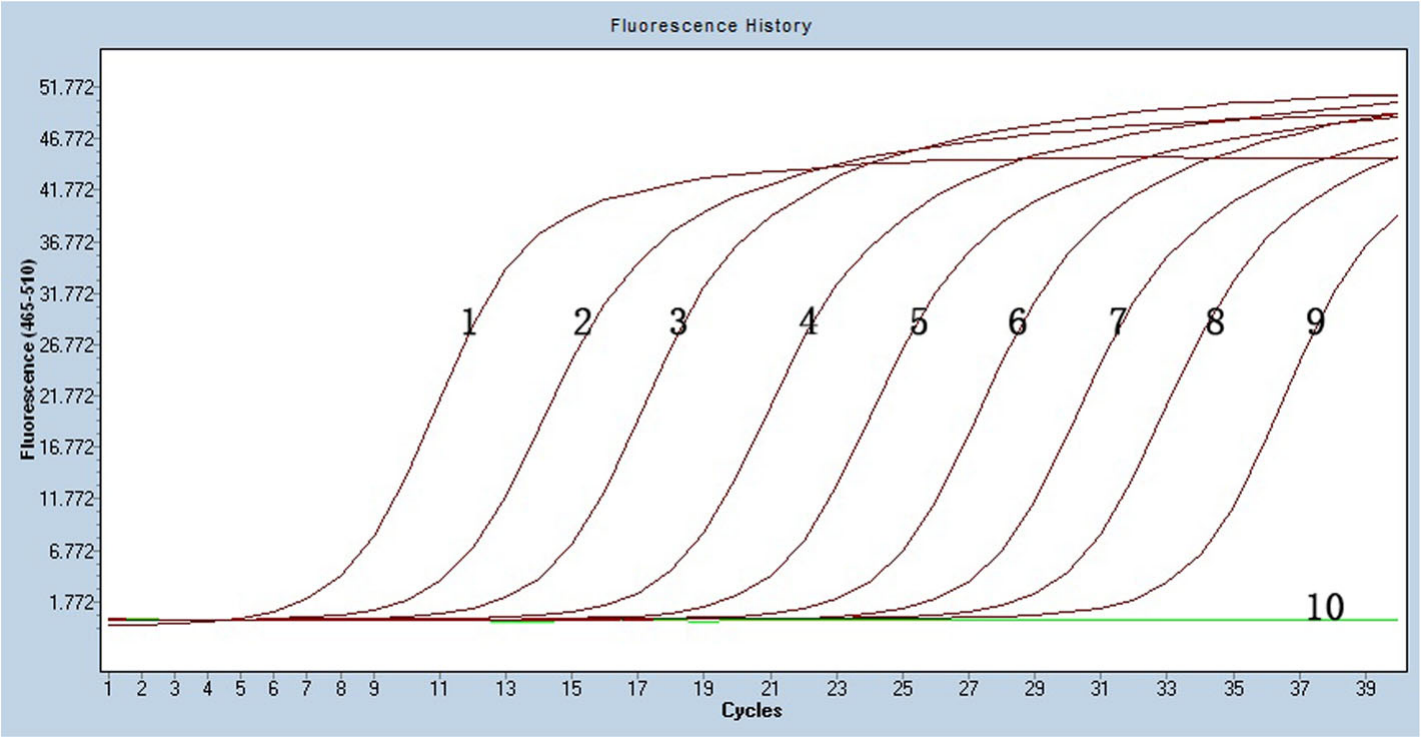
Sensitivity of the real-time RT-PCR assay. Templates of labeled 1 to 9 in these reactions with 10-fold serially diluted RNA ranged from 5.2 × 10^8^ to 5.2 × 10^0^ molecules per-reaction, and templates of labeled 10 was negative control (nuclease-free water)

**Fig. 3:**
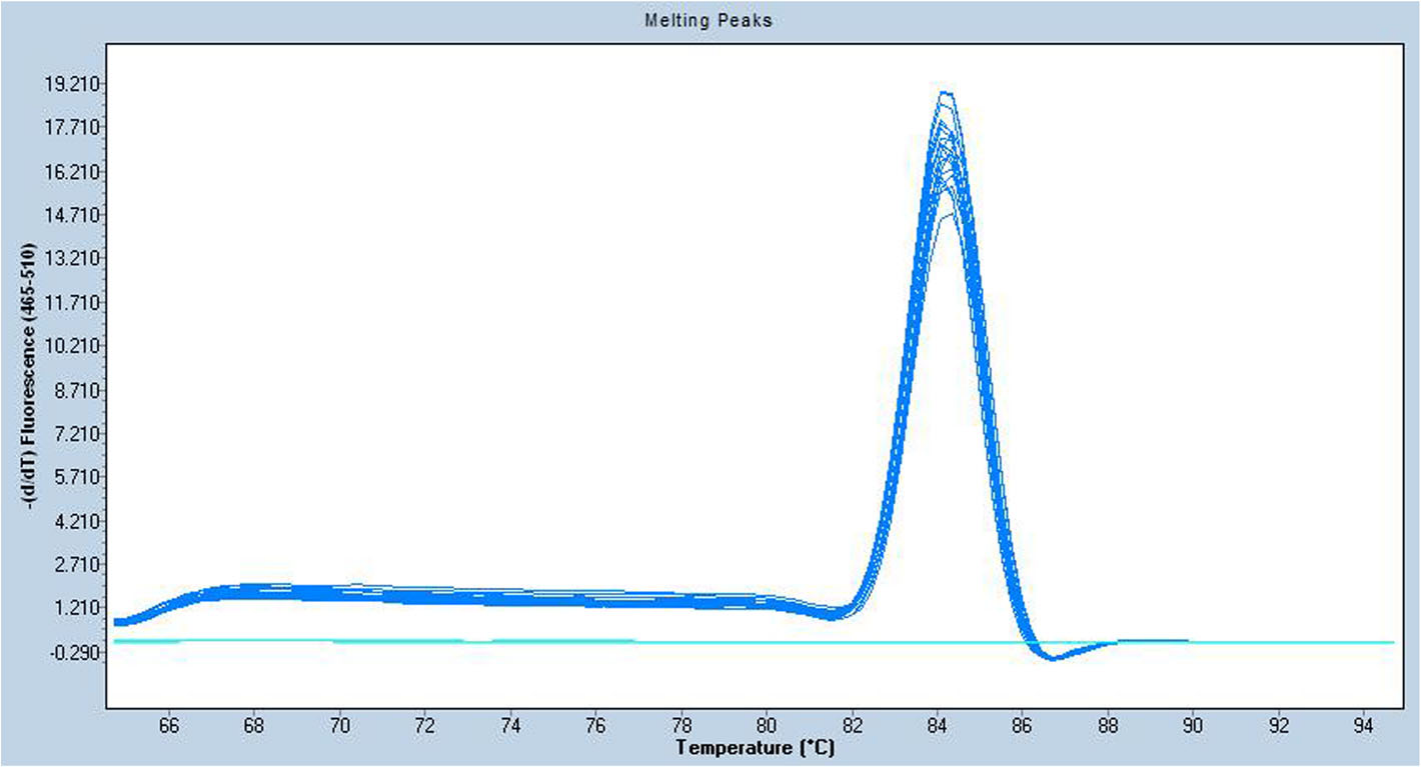
The dissociation plot of amplified BVDV-1 RNA molecular standard. Melting peaks of BVDV-1 RNA 10-fold serial dilutions and negative control. The positive samples showed an identical melting curve profile

The copy number of standard RNA transcribed from positive plasmid was 2.6 × 10^10^ RNA molecules/μL. Standard curve was generated by amplification of 10-fold-diluted molecular standard ranging from 5.2 × 10^8^ to 5.2 × 10^0^ RNA molecules per reaction. As is shown in Fig. 1, Ct values were representative of the cycle numbers, at which significantly increased fluorescence was firstly detected. According to the standard curve, the linear equation for the quantitative RT-PCR was y = − 3.4161x + 36.42. The slope of the standard curve was 3.416, and the efficiency of the reaction defined by 10^(− 1/slope)^ was 1.962, which was considered acceptable if it ranged from 1.7 to 2.2 [28, 29]. In addition, the correlation coefficient (*R*^*2*^ = 0.993) obtained by linear regression analysis demonstrated the consistency of the replicates. As for sensitivity of this method from the standard curve, the results showed that the lowest limit of this assay was 5.2 RNA molecules per reaction (Fig. 2). The melting curve analysis showed a single peak with an average melting temperature (Tm) of 84 ± 0.35 °C (Fig. 3).

### Specificity and reproducibility of the real-time RT-PCR

**Fig. 4:**
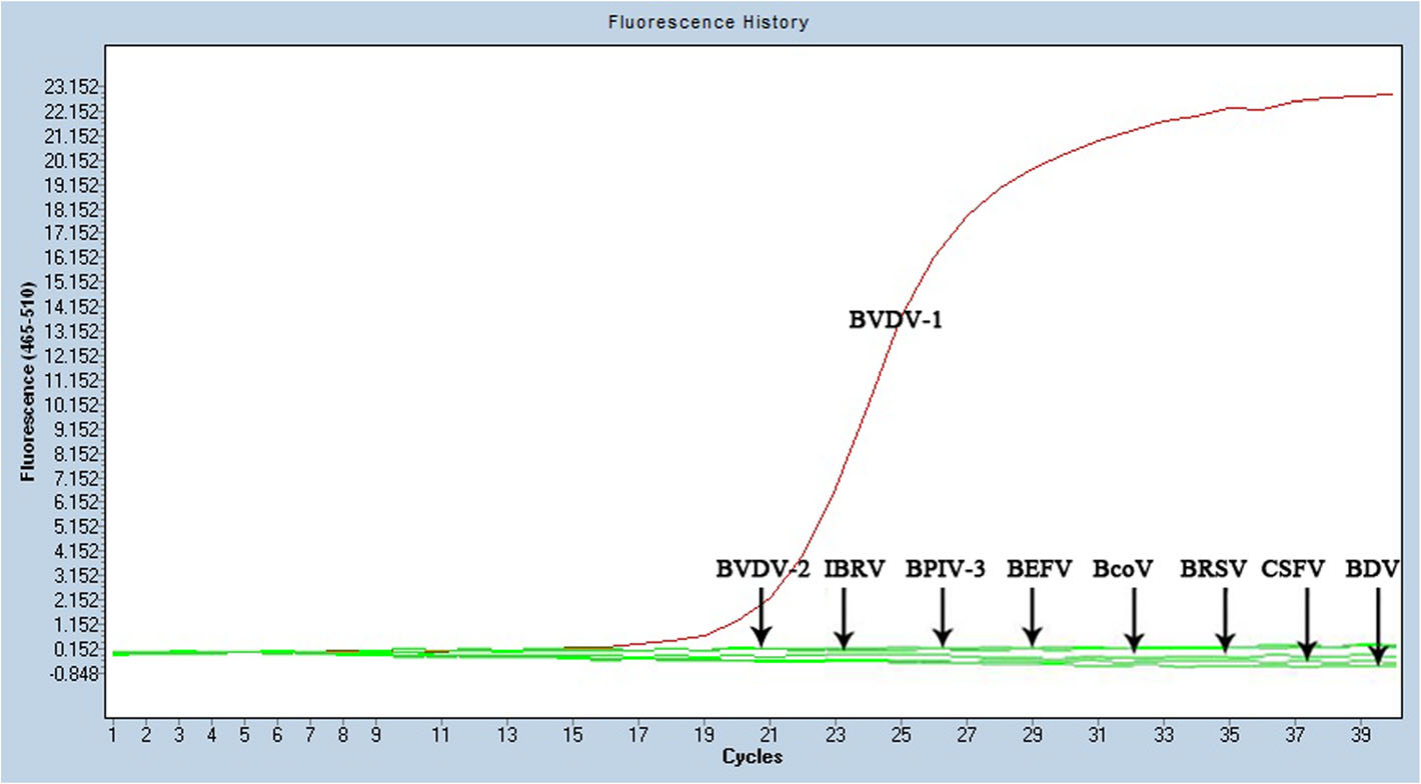
Specificity of the real-time RT-PCR assay. The specificity of the assay was assessed using other bovine viral pathogens and pestiviruses including IBRV, BPIV-3, BEFV, BcoV, BRSV*,* CSFV, BDV and BVDV-2

To assess the specificity, BVDV-1, BVDV-2 and other viral pathogens including IBRV, BPIV-3, BEFV, BRSV and BcoV as well as other pestiviruses (CFSV and BDV) were tested. As expected, no cross-reactions of BVDV-2, IBRV, BPIV-3, BEFV, BRSV, BcoV, CFSV and BDV were observed. Furthermore, no primer-dimers or non-specific amplifications were visible for negative samples (Fig. 4).

In order to evaluate the intra-assay and inter-assay reproducibility, nine 10-fold diluted RNA molecular standards were selected and repeated for three times. The results showed that the intra-assay mean ± SD of the CV was 1.46 ± 0.74% (variation range: 0.62–2.47%), and the inter-assay value was 1.34% ± 0.65% (variation range: 0.86–2.29%). Collectively, the established real-time RT-PCR assay was highly reproducible (Table 1).

**Table 1:** The variation coefficients of intra-assay and inter-assay reproducibility of the real-time RT-PCR

Sample	Intra-assay		Inter-assay	
	Mean CT ± SD	CV(%)	Mean CT ± SD	CV(%)
1	8.03 ± 0.19	2.37	8.75 ± 0.20	2.29
2	10.73 ± 0.25	2.33	10.52 ± 0.19	1.81
3	13.94 ± 0.20	1.43	14.11 ± 0.23	1.63
4	17.69 ± 0.11	0.62	17.71 ± 0.18	1.02
5	20.82 ± 0.16	0.77	20.65 ± 0.19	0.92
6	24.12 ± 0.28	1.16	24.32 ± 0.21	0.86
7	27.09 ± 0.33	1.22	27.94 ± 0.26	0.93
8	29.94 ± 0.23	0.77	30.11 ± 0.13	0.43
9	33.25 ± 0.82	2.47	34.35 ± 0.73	2.13

*CV* Coefficient of variation

### Performance of BVDV-1 real-time RT-PCR assay on aerosol specimen

A total of 169 aerosol samples collected from 6 dairy herds at different locations (Milking Parlour, Cowshed, Cattle playground) were simultaneously detected by RT-PCR and real-time RT-PCR approaches. The results of those assays showed that out of the 169 samples, 29 aerosol samples were positive by the real-time RT-PCR assay, while 15 specimens were positive using the RT-PCR assay (Table 2). Therefore, the positive rate detected by conventional RT-PCR, and real-time RT-PCR was 8.88% (15/169), and 17.16% (29/169), respectively. Overall, the detection rate of real-time RT-PCR was significantly higher than that of RT-PCR. In addition, to further confirm the assay performed on aerosol samples of transiently infected and persistently infected animals, RNA was extracted from aerosol specimens from known negative, transiently infected and persistently infected BVDV-1 animals. Table 3 shows that both transiently infected and persistently infected BVDV-1 aerosol specimens were positive. Additionally, the positive samples (*n* = 29) detected by real-time RT-PCR were tested by BVDV RPA-LFD, and a concordance rate of 100% was obtained between them (S. Table 1). Collectively, the results showed that the BVDV-1 real-time RT-PCR assay might be an ideal method for the detection of not only persistently infected but also transiently infected BVDV-1 in aerosol samples.

**Table 2:** Comparative performances of RT-PCR and Real-time RT-qPCR assays for detection of aerosol specimens

Herd No.	Size	PI positivity	Sero-positivity	Samples background	Number of samples	RT-PCR		Real-time qPCR	
						P	N	P	N
A	700	2.4%	37%	Milking parlour	7	1	6	3	4
				Cowshed	7	1	6	2	5
				Cattle playground	8	0	8	0	8
B	168	1.2%	24%	Milking parlour	12	1	11	1	11
				Cowshed	6	1	5	1	5
				Cattle playground	9	0	9	0	9
C	306	3.0%	43%	Milking parlour	12	1	11	3	9
				Cowshed	10	1	9	3	7
				Cattle playground	12	0	12	0	12
D	209	2.9%	27%	Milking parlour	9	0	9	1	8
				Cowshed	13	2	11	5	8
				Cattle playground	10	0	10	0	10
E	525	3.5%	69%	Milking parlour	10	3	7	5	5
				Cowshed	11	2	9	2	9
				Cattle playground	9	0	9	0	9
F	326	1.3%	45%	Milking parlour	8	1	7	2	6
				Cowshed	8	1	7	1	7
				Cattle playground	8	0	8	0	8
				Total	169	15	154	29	140

*P* Positive, *N* Negative

**Table 3:** Performances of Real-time RT-qPCR assays for detection of aerosol specimens collected from known BVDV-1 status-negatives, Transient infection (TI) and Persistent infection (PI) cattle

BVDV-1 status	Group	No. of cattle	No. of samples	Real-time RT-qPCR assays	
				Positive	Negative
TI	1	10	2	2	0
	2	10	2	2	0
	3	10	2	2	0
PI	1	10	2	2	0
	2	10	3	3	0
	3	10	2	2	0
Healthy	1	10	2	0	2
	2	10	2	0	2
	3	10	2	0	2
	Total	90	19	13	6

## Discussion

Emerging evidence suggests that airborne transmission is one of the major routes for the spread of viral diseases in animals, such as influenza a virus (IAV) [20, 30, 31], foot-and mouth disease virus (FMDV) [21, 22, 32], porcine reproductive and respiratory syndrome virus (PRRSV) [33, 34], porcine epidemic diarrhea virus (PEDV) [35], and bovine herpesvirus 1 (BHV-1) [36]. As for pestivirus infection, airborne transmission of CSFV and BVDV is feasible under experimental conditions although they have not been reported in field situations [37, 38], suggesting that airborne transmission may contribute to the spread of pestivirus infection.

Assays based on traditional RT-PCR have been applied to detect some airborne viruses [39]. However, these methods have their own weaknesses in terms of low sensitivity, and specificity as well as requirement of post-PCR processing steps. Real-time quantitative RT-PCR is suitable and widely accepted for the detection of a variety of pathogens because of its inherently quantitative nature, high sensitivity, specificity, and its rapidity. For BVDV, several real-time RT-PCR protocols have been developed for the detection of viral nucleic acid [40–44]. However, most of available real-time RT-PCR protocols are based on TaqMan assay, which greatly depends on proper primers and requires the design of long virus-specific probes. In the present study, we, for the first time, described a simple and economical SYBR Green-based RT-PCR assay for the detection of BVDV-1 in aerosol samples. BVDV-1 real-time RT-PCR primers were designed based on conserved 5′-UTR sequences of dominant BVDV-1 strains circulating in the world, especially in China to ensure the most effective detection coverage. Moreover, our results indicated that the newly developed method had high specificity, good reproducibility and low detection limit, which largely improved the detection efficiency of BVDV-1 airborne particles at low density.

To evaluate the validity of this method, an extensive performance on aerosol samples collected from different locations of dairy herds as well as control aerosol sampling were conducted. Our data showed that the method of aerosol detection could be a useful tool for the epidemiological investigation of non-invasive, detection of pathogens at the herd-level (a pooled sample). Nevertheless, diagnosis of viral aerosol remains a challenge due to several factors related to the lower concentrations of sample collection and smaller particle size. Furthermore, different factors are involved in the occurence and airborne transmission of BVDV [45]. Moreover, an effective aerosol collector is essential to collect virus-containing aerosols. In addition, the extraction efficiency of nucleic acid from viral particles is usually low, making it even more difficult to obtain enough RNA [46]. In our study, we chose 6 dairy herds with high persistent infection rate and seroprevalence without the use of vaccine. Indeed, there seemed to be no correlation between the seroprevalence in the individual farms (Table 2). Besides, the growth tube collector (GTC) system that utilized water-based condensation was supposed to be an efficient method to improve the collection efficiency, and aerosol sample suspension was enriched by ultracentrifugation to guarantee the amount of the maximum virus particle. Likewise, we further verified the BVDV-1 positive aerosol samples detected by the real-time RT-PCR using BVDV RPA-LFD. The results obtained from both methods were consistent, further indicating the validity and reliability of the test results. Although, RPA-LFD can also effectively detect BVDV-1 with the similar sensitivity, the cost of RPA-LFD is high, restricting its practical application.

It has been previously reported that the concentration of virus in aerosols is affected not only by environmental conditions (temperature, humidity and stocking density), but also by sample collection space. This could explain why aerosol samples collected from six cattle playgrounds all gave negative results. Moreover, in this study, we focused on detecting BVDV-1 directly from air samples instead of evaluating the concentrations of the virus transmission between cows. One factor that might contribute to this was that most of the samples were derived from open space, although the persistently infected cows were actively shedding virus. Therefore, more experimental studies are needed to investigate the viral load of BVDV-1 in aerosol in the future. Moreover, development of rapid and accurate diagnostic methods for BVDV detection in aerosol samples will help better understand the BVDV infection status of a herd, which plays a key role for the control of BVDV infection. Furthermore, several aerosol samples remained negative as cattle playground would be expected to have lower concentrations. However, despite the negative BVD status of all the aerosol samples collected from healthy cattle pens, only a limited number of samples were tested. Furthermore, accuracy (diagnostic sensitivity and specificity) of the test is further needed.

## Conclusions

A real-time RT-PCR approach based on SYBR Green was successfully developed for the rapid, sensitive and specific detection of BVDV-1 in aerosol samples. In addition, such approach probably provided an alternative for the prevalence investigation of BVDV-1 infection.

A SYBR Green based real-time RT-PCR assay was successfully developed for the rapid, sensitive and specific detection of BVDV-1 in aerosol samples. In addition, this assay probably provides an alternative for the prevalence investigation of BVDV-1 infection.

## Methods

### Viruses, cells and aerosol samples

BVDV-1/NADL strain preserved in our laboratory was used for preparation of template and standardization of this method. BVDV-2 HLJ-10 strain and other infectious bovine viral strains, including infectious bovine rhinotracheitis virus (IBRV), bovine parainfluenza virus type 3 (BPIV-3), bovine respiratory syncytial virus (BRSV), bovine ephemeral fever virus (BEFV) and bovine coronavirus (BcoV), were used for cross reactivity testing as previously described [47–49]. In addition, specific detection of other pestiviruses, such as classical swine fever virus (CFSV) and border disease virus (BDV), was performed using the synthesized 5ʹ-UTR gene fragment of corresponding genome. A total of 169 aerosol samples were collected from 6 dairy herds, with the prevalence of persistently infected animals was up to 1.2–3.5%, and the seroprevalence ranged from 24 to 69%, which was detected by BVD/MD P80-ELISA test kit (LABORATORIOS HIPRA S.A. Spain). Aerosol samples of various populations, both by geography or management (Milking Parlour, Cowshed, Cattle playground), as well as known BVDV status including 10 negative, transient and persistent infections (Table 3) were respectively collected with liquid bioaerosol samplers according to All-Glass Impinger(AGI) (Ace Glass Inc., Vineland, NJ) operation instructions. In brief, the AGI-30 impinger was installed and placed on three tangential nozzles with the height of 1.5 m above the ground, and it was operated with 10 mL of phosphate buffer saline (PBS) injected into the Proton sampler (S. Figure 1). The airflow rate of filtration was 12.5 L/min for 30 min [50], and aerosol samples obtained at different locations in dairy herds were transferred into 15-mL centrifuge tubes in a cold box and transported to the laboratory.

### Primer design and synthesis

A total of 69 sequences from different BVDV-1 lineages were obtained from NCBI GenBank database (http://www.ncbi.nlm.nih.gov/) (S. Table 2) and aligned using the Clustal W program (MEGA 5.0 software). Forward primers (5′-TGGTGAGTTCGTTGGATGGCTTAA-3′) and reverse primers (5′-CCCTATCAGGCTGTATTCGT-3′) were used for the amplification of 5′-UTR region of the BVDV-1 genome using real-time RT-PCR. Primers were designed with Primer Express 3.0 designer software and synthesized by TsingKe Biotech (Qingdao, China). The 5′-UTR region fragment (300 bp) of BVDV-1 was synthesized by Jierui (Shanghai, China) and cloned into pEASY-T3 cloning vector, which was used as the positive plasmid. The RNA molecular standard derived from the positive plasmid was prepared as previously described [47–49].

### Determination of standard curve, sensitivity and analysis of solution curve

In the present study, 10-fold serial dilutions of RNA molecular standard ranging from 5.2 × 10^8^–5.2 × 10^0^ RNA molecules per reaction were prepared for the standard curve. Real-time RT-PCR was performed on a Light Cycler 480 Real-Time PCR System using One Step TB Green® PremixScript™ RT-PCR kit (Takara Bio Inc., Japan). Briefly, after a reverse transcription at 42 °C for 5 min, and 95 °C for 10 s, amplifications were carried out with 40 cycles at a melting temperature of 95 °C for 5 s and an annealing temperature of 60 °C for 20 s, followed by a melting curve analysis under conditions as follows: 95 °C for 5 s, 65 °C for 60 s, and 95 °C for 15 s [51]. The standard curve was generated using 10-fold-serial dilutions of molecular standard and threshold cycle (Ct) values. The Ct values were compared between each of them, and the sensitivity of this method from the standard curve was determined. Moreover, the presence of non-specific PCR amplification could be ruled out by performing a melting curve analysis.

### Determination of specificity, and reproducibility of the assay

The specificity of the assay was assessed among other pestiviruses and viral pathogens of cattle including BVDV-2, CFSV, BDV, IBRV, BPIV-3, BRSV, BEFV and BcoV. In addition, the quality control of BVDV-2, CFSV, BDV, IBRV, BPIV-3, BRSV, BEFV and BcoV used in this study was conducted by common one-step PCR with corresponding primers that were listed in references [11, 52], and the PCR products was detected on 2% agarose gel to assure the quality and quantity of RNA. Finally, 5.2 × 10^4^ RNA molecules per-reaction and nuclease-free water were used as the positive control and negative control, respectively.

To evaluate the detection limit of the real-time RT-PCR assay, 5.2 × 10^8^ to 5.2 × 10^− 1^ RNA molecules per-reaction were assessed by using three replicates of each dilution, and the corresponding Ct values were used to make the standard curve for absolute quantification of BVDV-1 RNA. In diagnostic real-time PCR assays, it was customary to regard values results between Ct 35 and 40 as equivocal, while those above Ct 40 were regarded as negative [53].

To evaluate the reproducibility of the assay, molecular standards ranging from 5.2 × 10^8^ to 5.2 × 10^0^ RNA molecules per-reaction were assessed by real-time RT-PCR with three replicates of each dilution. Intra-assay reproducibility was evaluated by testing the standard deviation of each dilution and coefficients of variation (CVs), whereas the inter-assay reproducibility of each tested sample was calculated by repeating the experiment three times over an interval of 10 days.

### Performance of real-time RT-PCR, and RT-PCR assay on aerosol specimen

In order to verify the detection method, field-based BVDV aerosols and aerosol samples from known BVDV-1 negative, transiently infected and persistently infected housed individuals were used as negative and positive controls, respectively. Specifically, BVDV-1 negative herds were detected using every animal in the herd, with whole blood and nasal swab samples. The blood and nasal swab samples were tested by RT-PCR. The antibody of the blood samples was also detected by BVD/MD P80-ELISA test kit. All samples negative for BVDV-1 antigen and antibodies against BVD-1 virus were individually identified as non-BVDV infected herds. Transiently infected animals were collected from experimental cattle in Shandong Province, China and infected with BVDV-1. All blood samples and nasal swab samples of transiently infected animals (10 days post infection [dpi]) were determined positive by conventional RT-PCR and real-time RT-PCR, while transiently infected animals also can spread the infection during a brief viraemia (7–10 days long) [5]. To assess whether BVDV-1 positive dairy cattle were persistently infected animals, blood samples were collected at the interval of 3 weeks, and repeatedly detected by real-time RT-PCR. It would be considered as the persistently infected cattle once the detection of BVDV-1 gene was positive. Furthermore, the collected liquid was centrifuged at 4 °C, for 30 min at 10,000 g to remove bacteria and dust particles and then the aerosol sample suspension was ultracentrifuged at 4 °C, for 30 min at 100,000 g. The viral pellet was resuspended in 1 mL PBS (0.1 M, pH 7.2). Then the resuspended sample was used for RNA extraction with the QIAamp viral RNA minikit according to manufacturer’s instructions (Qiagen, Germany). The SYBR Green-based real-time RT-PCR method was conducted with the viral RNA extracted from aerosol samples. These results were analyzed by corresponding Ct values and melting curve. Additionally, the positive samples detected by real-time RT-PCR were confirmed by a rapid recombinase polymerase amplification in combination with a lateral flow dipstick (RPA-LFD) for BVDV to verify the accuracy and reliability of testing results [8].

Detection of aerosol samples was also performed by the conventional RT-PCR as previously described [52]. Sequences of PCR primers were as follows: forward primer 5′-ATGCCCATAGTAGGACTAGC-3′, and reverse primer 5′-CTCCATGTGCCATGTACAG-3′. Briefly, after an initial denaturation step at 95 °C for 5 min, the amplifications were carried out with 35 cycles at a melting temperature of 94 °C for 30 s, an annealing temperature of 54 °C for 30 s, and an extension temperature of 72 °C for 45 s, followed by an extra extension at 72 °C for 10 min. The size of the amplicon was 289 bp.

## Supplementary information

**Additional file 1: S. Figure 1.** Schematic of the air sampling apparatus. The schematic of the air sampling apparatus is not in proportion to air sampling device.

**Additional file 2: S. Table 1.** Further comparative performances of Real-time RT-qPCR and RPA-LFD assays for positive BVDV-1 nucleic acid aerosol samples detected by the real time RT-PCR. **S. Table 2.** The GenBank accession numbers of all sequences used for this study.

## Data Availability

The datasets generated and/or analysed during the current study were collected from the National Center for Biotechnology Information (NCBI) GenBank repository. The GenBank accession numbers of all sequences during this study were listed in the S.Table 2 (Supplementary file).

## Abbreviations

BVDV: Bovine viral diarrhea virus
CSFV: Classical swine fever virus
BDV: Border disease virus
5′-UTR: 5’untranslated region
ORF: Open reading frame
3′-UTR: 3′ untranslated region
BTM: Bulk tank milk
IBRV: Infectious bovine rhinotracheitis virus
BPIV-3: Bovine parainfluenza virus type 3
BRSV: Bovine respiratory syncytial virus
BEFV: Bovine ephemeral fever virus
BcoV: Bovine coronavirus
TI: Transient infection
PI: Persistent infection
PBS: Phosphate buffer sterile
CVs: Coefficients of variation
IAV: Influenza a virus
FMDV: Foot-and mouth disease virus
PRRSV: Porcine reproductive and respiratory syndrome virus
PEDV: Porcine epidemic diarrhea virus
BoHV-1: Bovine herpesvirus 1
