# Supplementary figures and images for: Detection of bovine viral diarrhea virus genotype 1 in aerosol by a real time RT-PCR assay
Source: BMC Vet Res. 2020 Apr 15;16:114. doi: 10.1186/s12917-020-02330-6 (PMC7159024; doi:10.1186/s12917-020-02330-6)

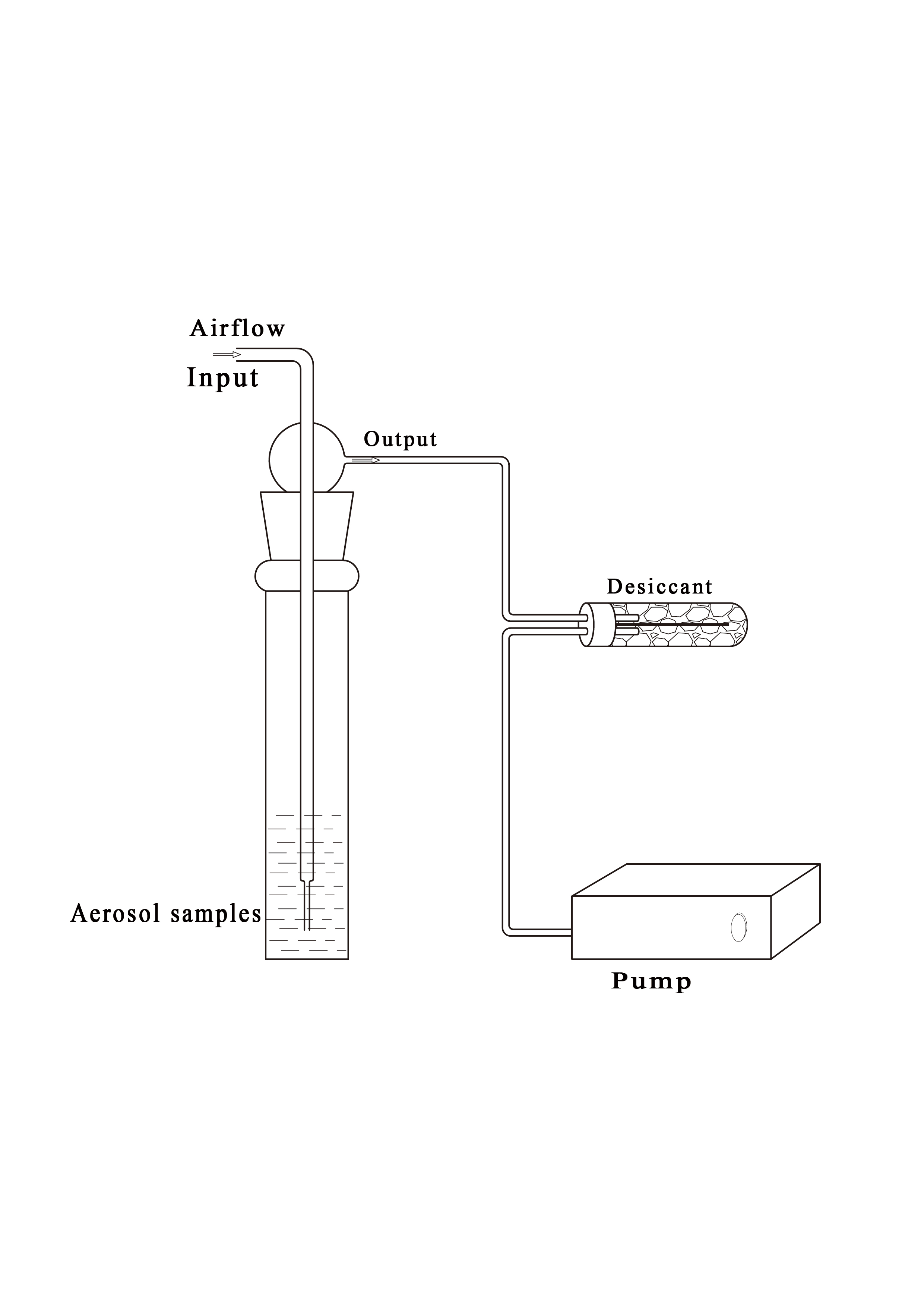

Supplement: Supplementary file 1 — Additional file 1: S. Figure 1. Schematic of the air sampling apparatus. The schematic of the air sampling apparatus is not in proportion to air sampling device. [file 12917_2020_2330_MOESM1_ESM.jpg]
