# Supplementary material for: Detection of bovine viral diarrhea virus genotype 1 in aerosol by a real time RT-PCR assay
Source: BMC Vet Res. 2020 Apr 15;16:114. doi: 10.1186/s12917-020-02330-6 (PMC7159024; doi:10.1186/s12917-020-02330-6)
Supplement: Supplementary file 2 — Additional file 2: S. Table 1. Further comparative performances of Real-time RT-qPCR and RPA-LFD assays for positive BVDV-1 nucleic acid aerosol samples detected by the real time RT-PCR. S. Table 2. The GenBank accession numbers of all sequences used for this study. [file 12917_2020_2330_MOESM2_ESM.docx]

**S. Table 1. Further comparative performances of Real-time RT-qPCR and RPA-LFD assays for positive BVDV-1 nucleic acid aerosol samples detected by the real time RT-PCR.**

| Herd No. | Number of  samples | Real-time qPCR | **RPA-LFD** | |
| --- | --- | --- | --- | --- |
|  |  | P | P | N |
| A | 5 | 5 | 5 | 0 |
| B | 2 | 2 | 2 | 0 |
| C | 6 | 6 | 6 | 0 |
| D | 6 | 6 | 6 | 0 |
| E | 7 | 7 | 7 | 0 |
| F | 3 | 3 | 3 | 0 |
|  | 29 | 29 | 29 |  |

**S. Table 2.**

| **The sequences used for this study** | |
| --- | --- |
| **GenBank**  **accession number** | **Strain** |
| AM749198.1 | VE/76/05 |
| AM749178.1 | TVM2/120/66 |
| AM749177.1 | PU/32/00 |
| AM749174.1 | MA/101/05 |
| AM749171.1 | ER/23/05 |
| AM749166.1 | LA/99/06 |
| AM749025.1 | UM/112/06 |
| AM748724.1 | Buffalo 113/03 |
| AM709624.1 | 05/15 |
| KF205290.1 | 58-09 |
| KF205281.1 | 21-12 |
| MK059454.1 | JSXLY1712 |
| KT833786.1 | 652TboUY/2014 |
| KF925520.1 | BJ1302 |
| JX966091.1 | TGL_ca_09 |
| JX966090.1 | ELV_ca_10 |
| JX966089.1 | AAB_nb_12 |
| JX966088.1 | RIB_ca_11 |
| FJ895328.1 | BR-UNESP-JAB 2 |
| JQ679457.1 | Ind S-18119 |
| EF683556.1 | 303 |
| EF683555.1 | ufms3 |
| GU385896.1 | BJ5(09) |
| LM994674.1 | SI/207/12 |
| LM994673.1 | UM/136/08 |
| LM994672.1 | CA/181/10 |
| LC054000.1 | IR-Isfahan-42 |
| LC053998.1 | Iran-Ahvaz-1 |
| KF434630.1 | KIRIKKALE90 |
| EU555287.1 | LTY |
| FJ621582.1 | Yili |
| FJ621580.1 | Shihezi 2 |
| FJ621577.1 | Hami 1 |
| FJ621575.1 | Akesu 1 |
| MN565878.1 | China/17142/2018 |
| MN417926.1 | T6-15-C |
| MN417885.1 | T5-50 |
| MN417826.1 | EN-19 |
| MG923949.1 | 42URU/2017 |
| MN442383.1 | HN1918 |
| MF803824.1 | BA1-2012 |
| MK982940.1 | ISF1979 |
| MN159203.1 | 3397CNESUY/2017 |
| MK622859.1 | 16M49 |
| MK381402.1 | 232-DA/18 |
| MK347344.1 | ET-94 |
| MK347342.1 | DO-48 |
| MK204920.1 | NMy1 |
| MK204905.1 | 810763 |
| MK170067.1 | BSC-1 |
| MG973218.1 | TR-Elz-Pst1 |
| MG913794.1 | TR-Erz-BV1 |
| MK127540.1 | Menofyia 2018 |
| MH673456.1 | TY8723 |
| MF803826.1 | GO-2013 |
| MH753471.1 | TR86 5' UTR |
| MG436781.1 | 12p |
| MF977722.1 | Ipixuna181_buf |
| MF124821.1 | LV/N928/15 |
| KY985233.1 | 98sv48 |
| KY985219.1 | 606sv48 |
| KY941184.1 | BVDV1/WB3/Serbia/2017 |
| KY865364.1 | HY-1 |
| KY865361.1 | WD-1 |
| KY886199.1 | S1118 |
| KY886197.1 | Mz/S1/14 |
| KY457411.1 | TNTIR16 |
| KY457410.1 | TNCBE16 |
| MG323525.1 | FS1426 |
